# Effect of prebiotic intake on gut microbiota, intestinal permeability and glycemic control in children with type 1 diabetes: study protocol for a randomized controlled trial

**DOI:** 10.1186/s13063-016-1486-y

**Published:** 2016-07-26

**Authors:** Josephine Ho, Raylene A. Reimer, Manpreet Doulla, Carol Huang

**Affiliations:** 1 Alberta Children’s Hospital, 2888 Shaganappi Trail NW, Calgary, Alberta T3B 6A8 Canada; 2 Faculty of Kinesiology, University of Calgary, 2500 University Drive, Calgary, Alberta T2N 1N4 Canada

**Keywords:** Type 1 diabetes, Child, Prebiotics, Gut microbiota, Intestinal permeability

## Abstract

**Background:**

The gut microbiome is increasingly recognized as a contributor to disease states. Patients with type 1 diabetes (DM1) have distinct gut microbiota in comparison to non-diabetic individuals, and it has been linked to changes in intestinal permeability, inflammation and insulin resistance. Prebiotics are non-digestible carbohydrates that alter gut microbiota and could potentially improve glycemic control in children with DM1. This pilot study aims to determine the feasibility of a 12-week dietary intervention with prebiotics in children with DM1.

**Methods/design:**

This pilot study is a single-centre, randomized, double-blind, placebo-controlled trial in children aged 8 to 17 years with DM1 for at least one year. Participants will be randomized to receive either placebo (maltodextrin 3.3 g orally/day) or prebiotics (oligofructose-enriched inulin 8 g orally/day; Synergy1, Beneo, Mannheim, Germany). Measures to be assessed at baseline, 3 months and 6 months include: anthropometric measures, insulin doses/regimens, frequency of diabetic ketoacidosis, frequency of severe hypoglycemia, average number of episodes of hypoglycemia per week, serum C-peptide, HbA1c, serum inflammatory markers (IL-6, IFN-gamma, TNF-alpha, and IL-10), GLP-1 and GLP-2, intestinal permeability using urine assessment after ingestion of lactulose, mannitol and 3-O-methylglucose, and stool sample collection for gut microbiota profiling.

**Discussion:**

This is a novel pilot study designed to test feasibility for a fully powered study. We hypothesize that consumption of prebiotics will alter gut microbiota and intestinal permeability, leading to improved glycemic control. Prebiotics are a potentially novel, inexpensive, low-risk treatment addition for DM1 that may improve glycemic control by changes in gut microbiota, gut permeability and inflammation.

**Trial registration:**

ClinicalTrials.gov: NCT02442544. Registered on 10 March 2015.

## Background

The gut microbiome plays a key role in health but is increasingly recognized as a contributor to various disease states when an imbalance or dysbiosis occurs. Both animal and human studies found a difference in microbial composition between those that develop type 1 diabetes from those that did not develop diabetes [1]. In animal studies, interventions that change the gut microbiota can alter immune response and can play a role in the development of type 1 diabetes [2]. In children with type 1 diabetes (DM1), alterations in gut microbiota have been identified [3–6]. For example, bifidobacteria have previously been found to negatively correlate with beta-cell autoimmunity in Finnish children with diabetes-related autoantibodies [3] and in Spanish children with DM1 [6]. *Faecalibacterium prausnitzii*, a butyrate-producing bacterium, has been shown to have some anti-inflammatory effects and has been found to be decreased in children with diabetes-related autoantibodies [3].

Prebiotics are defined as selectively fermented ingredients that result in specific changes in the composition and/or activity of the gastrointestinal microbiota, thus conferring benefit(s) upon host health [7]. Prebiotics have been shown to increase the abundance of both bifidobacteria and *Faecalibacterium prausnitzii* [8] and, therefore, may help correct defects in the gut microbial environment associated with DM1 development and progression. Beyond reducing dysbiosis in the gut microbial environment, prebiotics have also been shown to improve glucose tolerance via mechanisms that likely include enhanced production and secretion of the incretin glucagon-like peptide 1 (GLP-1) [9]. Indeed, prebiotics improved hemoglobin A1c (HbA1c), postprandial glycemic excursion and inflammatory markers in patients with type 2 diabetes [9, 10]. To date however, there have been no studies examining the effect of using prebiotics to alter gut microbiota and intestinal permeability in children with DM1 and whether such changes can improve glycemic control.

In addition to increased incretin production, prebiotics may improve glycemia through its action on intestinal mucosal barrier function and gut microbiota [11–14]. Both animal and human studies have linked gut microbiota to metabolic dysregulation. Patients with diabetes have distinct gut microbiota in comparison to healthy individuals [3, 4], with a higher gram-negative to gram-positive bacterial ratio and a lower abundance of bifidobacteria, an important microbial population with many health benefits [1, 15, 16].

In animal studies, diabetes is also associated with increased gut permeability, allowing bacterial lipopolysaccharides (LPS) from gram-negative bacteria to translocate into the systemic circulation, causing metabolic endotoxemia, triggering pro-inflammatory cytokine secretion and insulin resistance [17–19]. Animal studies showed that prebiotic treatment dose-dependently increases bifidobacteria [20], reduces gut permeability and endotoxemia [21, 22] and improves glucose tolerance [23]. Indeed, bifidobacteria abundance negatively correlates with serum LPS, fasting insulin and glucose [21]. Prebiotics also enhance GLP-2 production, which restores tight junction protein expression and reduces gut permeability [18]. Hence, prebiotics may improve glucose homeostasis by two separate mechanisms: (1) up-regulating GLP-1 to improve beta-cell mass and function, and (2) altering gut microbiota and permeability to a less pro-inflammatory phenotype, improving insulin sensitivity. In humans, bacterial endotoxin activity has been shown to be associated with insulin resistance [24]. However, direct manipulation of gut microbiota in humans and its impact on gut permeability and glycemic control has not been studied, and whether these mechanisms also operate in humans is unknown.

Prebiotics are a potentially novel, inexpensive, low-risk treatment addition for DM1 that may improve glycemic control by changes in gut microbiota, gut permeability and inflammation. This pilot study will provide critical proof of concept data for future trials looking at the efficacy of using prebiotics as an adjunct in the management of DM1 to improve glycemic control.

The primary objective of this study is to determine the effect of a 12-week dietary intervention with 8 g/day of prebiotic intake compared to placebo on glycemic control as measured by HbA1c in children diagnosed with DM1 for at least one year. The secondary objective is to determine the gut microbiota composition in children diagnosed with DM1 consuming prebiotic versus placebo. Further, we aim to examine the differences in gut permeability, serum inflammatory markers, glucagon-like peptide 1 (GLP-1) and GLP-2 and C-peptide in children diagnosed with DM1 consuming prebiotics versus placebo and to assess for diabetes-related adverse reactions (i.e. severe hypoglycemia and diabetic ketoacidosis) associated with use of prebiotics in children with DM1.

## Methods/design

### Design

**Fig. 1:**
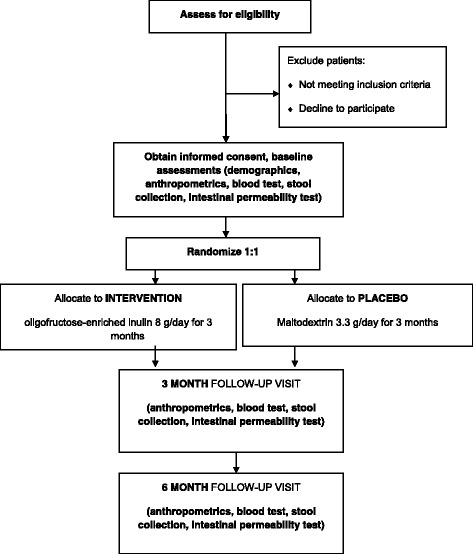
CONSORT flow diagram

This pilot study is a single-centre, randomized, double-blind, placebo-controlled trial of prebiotic treatment on gut microbiota, intestinal permeability and glycemic control in children aged 8 to 17 years who have had DM1 for at least one year. A schematic of the study design is given in Fig. 1.

### Centre and patient selection

Patients will be recruited from the diabetes clinic at the Alberta Children’s Hospital located in Calgary, Alberta, Canada. This is a tertiary care hospital that provides diabetes care to approximately 900 children with DM1. The inclusion and exclusion criteria are outlined in Table 1.

**Table 1:** Participant inclusion and exclusion criteria

Inclusion criteria	Exclusion criteria
• Age 8 years to 17 years (inclusive)	• Any hemoglobin A1c greater than 10 % in the previous 6 months
• Diagnosed with type 1 diabetes for at least 12 months	• Chronic medical condition that could affect gut microbiota (examples: Crohn’s disease, cystic fibrosis, irritable bowel syndrome, etc.)
• Followed at the Alberta Children’s Hospital diabetes clinic (Calgary, Alberta, Canada)	• Receiving medications or supplements that could affect gut microbiota (examples: antibiotics, probiotics, prebiotics, laxatives, etc.)
• Parent or legal representative of the patient is willing to give consent for the study and child assents	• Positive celiac disease screen

### Patient recruitment

Patients will be approached during their regular diabetes clinic visits to participate in this study. Posters and information pamphlets will be available so that families can contact the research team if they are interested in participating. Patients will be assessed for eligibility and written informed consent, and where appropriate, assent will be obtained for all study participants.

### Randomization

Participants will be randomized via computer-generated numbers to intervention or placebo control in a 1:1 ratio. The intervention and placebo groups will not be matched (e.g. age, duration of T1DM, etc.). An investigator not involved in running the study will prepare the randomization sequences and have the random allocation sequence in numbered envelopes in a secure location that will be opened only once a participant has consented and enrolled in the study. The participants, trial coordinator and research staff who enroll the patients will remain blinded to the participants’ group assignment. Participants will be asked at the end of the study to which group they believed they were assigned.

### Intervention

Participants will be randomized to receive either placebo (maltodextrin 3.3 g orally/day; Agenamalt 20.222, Agrana Starch, Konstanz, Germany) or prebiotic (oligofructose-enriched inulin 8 g orally/day; Synergy1, Beneo, Mannheim, Germany). Inulin and oligofructose are approved as food ingredients in Canada and have been used previously in clinical trials [8, 25]. Both the prebiotic and placebo will be provided to participants in powder form in identical foil pre-weighed packets. Participants will be instructed to mix the packet with 250 mL of water until dissolved and to drink it 15–20 minutes prior to their evening meal. For the first 2 weeks, participants will be asked to only take half of the dose in order to minimize gastrointestinal side effects, and then they will take the full dose for the remaining 10 weeks.

Participants will be asked to record any diabetes-related or gastrointestinal adverse reactions, i.e. frequency of mild hypoglycemia (symptoms of hypoglycemia with a blood glucose less than 4 mmol/L and able to self-treat with oral rapid-acting carbohydrate), severe hypoglycemia (symptoms of hypoglycemia with a blood glucose less than 4 mmol/L but requires assistance with treatment due to decreased level of consciousness or has a seizure), diabetic ketoacidosis or flatulence. At the end of the 12 weeks, participants will be asked to return any remaining packets of placebo or prebiotic in order to assess for compliance. Telephone contact from a member of the research team will occur monthly to encourage compliance and recording of adverse reactions.

### Data collection

Study visits will be coordinated with diabetes clinic visits; the data to be collected are outlined in Table 2. Demographic information will be collected at baseline. Anthropometric measures and assessment of insulin regimens, frequency of diabetic ketoacidosis (DKA) in previous 3 months, frequency of severe hypoglycemia in previous 3 months and average number of mild hypoglycemic episodes per week in the preceding 3 months will be assessed at baseline, 3 months and 6 months.

**Table 2:**
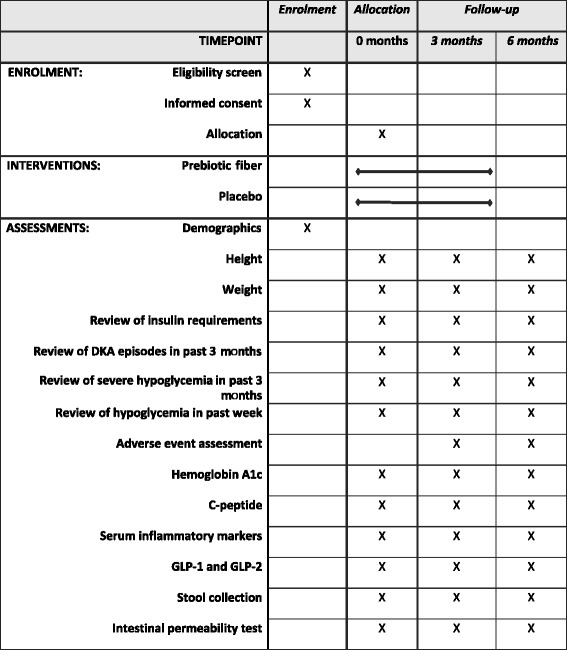
Study procedures and data collection

#### Glycemic control, inflammatory markers, GLP-1 and GLP-2

Blood will be collected at baseline, 3 months and 6 months for serum C-peptide, HbA1c, serum inflammatory markers (IL-6, IFN-gamma, TNF-alpha and IL-10), GLP-1 and GLP-2. Serum will be sent to Calgary Laboratory Services (Calgary, AB, Canada) for measurement of HbA1c by turbidimetric inhibition immunoassay (Roche Integra 800 CTS, Basel, Switzerland) and serum C-peptide by chemiluminescent assay (Siemens Immulite 2000, Erlangen, Germany). Serum inflammatory markers will be analysed by Milliplex Human Cytokine Magnetic Bead Multiplex kits (Millipore, St. Charles, MO, USA). GLP-1 and GLP-2 will be measured with ELISA kits (Millipore, St. Charles, MO, USA).

#### Intestinal permeability

Intestinal permeability will be assessed at baseline, 3 months and 6 months. Participants will consume a regular evening meal and then 3 hours later, prior to bedtime, drink a solution containing lactulose (5 g), mannitol (2 g) and 3-O-methylglucose (5 g) in 200 mL of water (Biosource, Canada). All urine for the following 12 hours will be collected with 5 mL thymol in the storage container for preservation, and stored frozen. High-performance liquid chromatography (HPLC) will be used to analyse urine samples for the lactulose, mannitol and 3-O-methylglucose content. Urine samples will be filtered through a 0.4-μm filter and diluted as necessary. Samples will be deionized and injected into an ion exchange column and eluted with NaOH at a flow rate of 0.4 mL/min with concentrations ranging from 400 to 600 mM. Peaks will be detected using pulse and amperometric detection in a Dionex HPLC. The fraction of the ingested dose recovered in the urine sample will be calculated and compared between the two groups [26, 27].

#### Gut microbial profiling

Stool samples will be collected at baseline, 3 months and 6 months as previously described [14, 20]. Participants will be provided with a Bristol stool chart and asked to mark down the type of stool (type 1 to type 7). Participants will be provided a stool collection kit and will be instructed on proper methods for stool collection. One tablespoon of stool will be placed in a pre-labelled sterile conical tube, placed in a biohazard bag and stored in the home freezer (–20 °C). Participants will bring samples to the laboratory up to 3 days from collection on ice, and then the samples will be stored in the laboratory freezer at –80 °C until analysed. Total bacterial DNA will be extracted from stool samples using the FastDNA Spin Kit for Feces (MP Biomedicals, Lachine, QC, Canada) followed by ethanol precipitation purification. DNA will be quantified using Qubit dsDNA assay (Promega, Madison, WI, USA) and diluted to 5 ng/μL concentration. Microbial composition will be determined as per our previously published protocol [28] following Illumina’s 16S rRNA amplicon sequencing protocol on the MiSeq platform (Illumina, San Diego, CA, USA). The V3 and V4 regions of the 16S rRNA gene will be amplified using 2.5 μL (5 ng/μL) microbial DNA, 5 μL (1 μM) of gene-specific primers and 12.5 μL 2x KAPA HiFi Hotstart Ready Mix (KAPA Biosystems, Boston, MA, USA). Following amplification, the PCR product will be purified (Ampure XP beads, Beckman Coulter, Mississauga, ON, Canada) and the amplicon size confirmed. Dual-indexed barcodes will be attached to amplicon targets in a second PCR stage. The final PCR product will be purified (Ampure XP beads, Beckman Coulter, Mississauga, ON, Canada) and quantified using the Qubit dsDNA assay (Promega). Amplicon size will be assessed using a D1000 TapeStation (Agilent Technologies) assay and samples normalized to 4 nM using 10 mM Tris pH 8.5. After pooling barcoded libraries, samples will be denatured and diluted to a final concentration of 4 pM, and a final product containing 10 % PhiX will undergo dual-indexed paired 300 bp sequencing on the MiSeq using Reagent kit v3 (Illumina). Paired-end reads will be merged using PEAR (Paired-End read merger) [29], and data analysis will be performed using the Quantitative Insights into Microbial Ecology (QIIME) pipeline version 1.9.1 [30]. Operational taxonomic units (OTUs) will be picked using UCLUST [31] with a 97 % sequence identity threshold followed by taxonomy assignment using the latest Greengenes database (http://greengenes.secondgenome.com). To evaluate beta diversity, principal coordinate analysis (PCoA) on weighted UniFrac distances will be performed on all OTUs using QIIME. Alpha diversity will be measured by calculating the Shannon index, Simpson index and Chao1 metrics using QIIME [30]. A false discovery rate will be used to control for type 1 error. Given that 16S rRNA sequencing generates relative abundance data, we will also quantify the abundance of select microbial groups (e.g. *F. prausnitzii*, *Bifidobacterium* spp., total bacteria) with quantitative PCR according to our previous work [14, 20] using the Bio-Rad iCycler (Bio-Rad Inc., Mississauga, ON) and group-specific primers.

### Sample size

#### Internal pilot

The initial pilot study will aim to test the feasibility of a fully powered studied in children with type 1 diabetes. To test feasibility in this pilot study, we will enroll 15 subjects per arm. If we assume a drop-out rate of 20 % (12 subjects completing the study in each arm), power of 80 %, and alpha of 0.05 and a standard deviation of 1.3 for HbA1c, then with the pilot study we would be able to detect a mean difference in the absolute HbA1c of 1.5 between the placebo and prebiotic groups.

#### Full trial

For the full study, the sample size was calculated for a two-sided *t* test comparing two independent samples. Based on previous follow-up data from the Alberta Children’s Hospital Diabetes Clinic, the mean score of HbA1C at baseline in both groups will be estimated to be 8.4 with a standard deviation of 1.3 for each group. A clinically significant change in absolute HbA1c of 0.5 will be used, as this threshold has been used in previous drug trials to demonstrate effectiveness in diabetes. For a power of 80 % and an alpha of 0.05, the number of subjects needed per arm of the study is 107. If we assume a drop-out rate of 20 %, then approximately 135 subjects per arm of the study will be required in a fully powered study.

### Analysis

Analysis will be performed using SPSS 22.0 software (IBM, New York, USA). Results will be considered statistically significant if *p* ≤ 0.05. Baseline descriptive data between the control and intervention group will be compared using chi-square for categorical variables and *t* tests for continuous variables. The primary outcome of HbA1c will be expressed as mean HbA1c values with standard deviations, and a two-sided *t* test will be used to compare HbA1c between placebo and prebiotic groups. Differences between the placebo and prebiotic groups’ C-peptide, inflammatory markers (IL-6, IFN-gamma, TNF-alpha, and IL-10), GLP-1, GLP-2 and intestinal permeability will be compared using a two-sided *t* test. Adverse events will be presented as the proportion of each event seen in each group (placebo and prebiotic). An intent-to-treat analysis will be used with the last data point carried forward for missing data. A secondary analysis will also be performed on a per-protocol basis with all subjects who have completed the intervention and are reported to be compliant with ≥80 % of the prebiotic or placebo packets consumed.

## Discussion

DM1 in children is a chronic disease with the current mainstay of treatment being subcutaneous insulin administration multiple times a day, frequent capillary glucose monitoring and monitoring/counting carbohydrate intake at all meals and snacks. The management regimen in DM1 is invasive and labor intensive and may not always result in optimal glycemic control. Therefore, the addition of a simple, oral supplement such as a prebiotic to improve glycemic control would be very beneficial in this population.

Based on animal and human studies to date [9], we anticipate that a change in gut microbiota, gut permeability and inflammatory markers could occur over the course of several weeks of prebiotic supplementation. Hence, we anticipate that changes in these parameters will be detectable at the 3-month follow-up. The change in HbA1c will likely lag behind — as the life span of glycosylated red blood cells is 3 months, any improvement in glycemic control as a result of the 3-month prebiotic treatment may not be fully reflected at the 3-month follow-up, but may only become detectable at the 6-month follow-up. We will reassess all these parameters at 6 months to determine the persistence of effects and whether continued consumption of prebiotics is necessary to maintain the changes in gut microbiota, gut permeability and inflammatory markers observed.

Kellow et al. [9] published a systematic review on the metabolic benefits of prebiotics in human randomized controlled trials. Meta-analysis indicated a statistically significant effect of prebiotics on reduction in post-prandial glucose and post-prandial insulin levels. Conflicting results were seen in studies looking at fasting glucose, fasting insulin and HbA1c. No pediatric studies or populations with DM1 were identified for inclusion in this systematic review.

Murri et al. showed that children with established DM1 have different gut microbiota from children without T1DM [6]. In addition to changes in the gut microbiota, altered gut permeability may also contribute to the pathogenesis of type 1 diabetes, as increased ‘gut leakiness’ has been shown in animal models preceding the development of diabetes [26]. It is hypothesized that the increased gut permeability allowed continued exposure to antigens that contribute to aspects of the immune dysregulation observed in DM1 [1]. Importantly, prebiotics have been shown to reduce gut permeability through a mechanism that involves GLP-2, a gut trophic factor [18]. In this current study, we aim to determine if treatment with prebiotics can decrease gut permeability, which will decrease endotoxemia and reduce insulin resistance. Improved insulin resistance may lead to improved glycemic control. In future studies, the role of prebiotics in altering disease progression in pre-diabetes will be examined.

This study will provide valuable information on whether the consumption of a prebiotic will alter gut microbiota and intestinal permeability in children with type 1 diabetes. This could potentially lead to improved glycemic control and adjunctive therapy that is inexpensive and easy to administer.

## Trial status

The protocol has been approved by the Conjoint Health Research Ethics Board, University of Calgary (Calgary, Alberta, Canada) 10 June 2015 and has been registered at ClinicalTrials.gov on 10 March 2015 (NCT02442544). Recruitment has just commenced at Alberta Children’s Hospital.

## Abbreviations

GLP, glucagon-like peptide; HbA1c, hemoglobin A1c; IFN, interferon; IL, interleukin; TNF, tumor necrosis factor.
